# Molecular Evidence of *Hemolivia mauritanica*, *Ehrlichia* spp. and the Endosymbiont *Candidatus* Midichloria Mitochondrii in *Hyalomma aegyptium* Infesting *Testudo graeca* Tortoises from Doha, Qatar

**DOI:** 10.3390/ani11010030

**Published:** 2020-12-26

**Authors:** Patrícia F. Barradas, Clara Lima, Luís Cardoso, Irina Amorim, Fátima Gärtner, João R. Mesquita

**Affiliations:** 1 Institute of Biomedical Sciences Abel Salazar (ICBAS), University of Porto, 4050-313 Porto, Portugal; patriciaferreirabarradas@gmail.com (P.F.B.); iamorim@ipatimup.pt (I.A.); fgartner@ipatimup.pt (F.G.); 2 Epidemiology Research Unit (EPIUnit), Instituto de Saúde Pública da Universidade do Porto, 4050-091 Porto, Portugal; 3 Department of Biological Sciences, Microbiology Laboratory, Faculty of Pharmacy, University of Porto, 4050-313 Porto, Portugal; claramlima@gmail.com; 4 Department of Veterinary Sciences, and Animal and Veterinary Research Centre (CECAV), University of Trás-os-Montes e Alto Douro, 5000-801 Vila Real, Portugal; lcardoso@utad.pt; 5 Institute for Research and Innovation in Health (i3S), University of Porto, 4200-135 Porto, Portugal; 6 Institute of Molecular Pathology and Immunology of the University of Porto (IPATIMUP), 4200-135 Porto, Portugal; 7 Department of Veterinary Clinics, ICBAS-UP, Rua de Jorge Viterbo Ferreira 228, 4050-313 Porto, Portugal

**Keywords:** endosymbionts, *Hemolivia*, surveillance, tortoises, tick-borne pathogens, ticks

## Abstract

**Simple Summary:**

Due to the veterinary and medical importance of pathogens transmitted by *Hyalomma aegyptium*, we tested ticks removed from *Testudo graeca* tortoises for the presence of *Anaplasma*, *Ehrlichia*, *Hemolivia*, *Babesia* and *Hepatozoon*. Forty-three percent of the examined adult ticks were infected with at least one agent. The most prevalent agent identified was *Hemolivia mauritanica* (28.6%), followed by *Candidatus* Midichloria mitochondrii (9.5%) and *Ehrlichia* spp. (4.7%). Our study reported for the first time *H. mauritanica*, *Ehrlichia* spp. and *Candidatus* M. mitochondrii in *H. aegyptium* ticks collected from pet spur-thighed tortoises, in Qatar, providing data that adds to the geographical extension of these agents.

**Abstract:**

Tick-borne agents constitute a growing concern for human and animal health worldwide. *Hyalomma aegyptium* is a hard tick with a three-host life cycle, whose main hosts for adults are Palearctic tortoises of genus *Testudo*. Nevertheless, immature ticks can feed on a variety of hosts, representing an important eco-epidemiological issue regarding *H. aegyptium* pathogens circulation. *Hyalomma aegyptium* ticks are vectors and/or reservoirs of various pathogenic agents, such as *Ehrlichia*, *Anaplasma*, *Babesia* and *Hepatozoon/Hemolivia*. *Ehrlichia* and *Anaplasma* are emergent tick-borne bacteria with a worldwide distribution and zoonotic potential, responsible for diseases that cause clinical manifestations that grade from acute febrile illness to a fulminant disease characterized by multi-organ system failure, depending on the species. *Babesia* and *Hepatozoon/Hemolivia* are tick-borne parasites with increasing importance in multiple species. *Testudo graeca* tortoises acquired in a large animal market in Doha, Qatar, were screened for a panel of tick-borne pathogens by conventional PCR followed by bidirectional sequencing. The most prevalent agent identified in ticks was *Hemolivia mauritanica* (28.6%), followed by *Candidatus* Midichloria mitochondrii (9.5%) and *Ehrlichia* spp. (4.7%). All samples were negative for *Babesia* spp. and *Hepatozoon* spp. Overall, 43% of the examined adult ticks were infected with at least one agent. Only 4.7% of the ticks appeared to be simultaneously infected with two agents, i.e., *Ehrlichia* spp. and *H. mauritanica*. This is the first detection of *H. mauritanica*, *Ehrlichia* spp. and *Candidatus* M. mitochondrii in *H. aegyptium* ticks collected from pet spur-thighed tortoises, in Qatar, a fact which adds to the geographical extension of these agents. The international trade of *Testudo* tortoises carrying ticks infected with pathogens of veterinary and medical importance deserves strict control, in order to reduce potential exotic diseases.

## 1. Introduction

Ticks are known as important vectors of many viral, bacterial and protozoan infectious microorganisms capable of producing disease in both humans and animals [1]. As hematophagous arthropods, while taking a blood meal, they can transmit pathogens to susceptible hosts, supporting the enzootic cycles of many infectious agents in various ecosystems and being regarded as major human and veterinary public health problems [2]. Nevertheless, these arthropods also harbor intracellular bacteria that are apparently not detrimental to humans, animals or even to ticks themselves. Symbionts, such as *Candidatus* Midichloria mitochondrii, are obligately intracellular bacteria, and in some cases are closely associated with the presence of known pathogens, such as *Rickettsia parkeri* [3]. Symbiotic, commensal and pathogenic microorganisms harbored by ticks can positively influence pathogen transmission or interfere with their maintenance in the tick [4]. For example, *Coxiella*-like endosymbionts seem to impair the transmission of *Ehrlichia chaffeensis* by *Amblyomma* ticks [5], whereas the presence of *Francisella* sp. endosymbionts increases the colonization success of pathogenic *Francisella novicida* in *Dermacentor andersoni* ticks [6].

*Hyalomma aegyptium is a* three-host life cycle hard tick endemic in North Africa, Balkan countries, the Middle East, Caucasus, Central Asia, Afghanistan and Pakistan, whose adult stage main hosts are Palearctic tortoises of the genus *Testudo* [7,8,9]. However, adult ticks, together with the less host-specific nymphs and larvae, also feed on various vertebrates, such as domestic animals (dogs, cattle, pigs, horses), wild animals (birds, boar, deer, foxes, jackals, hamsters, hares, hedgehogs, mustelids, squirrels) and humans [10,11,12,13,14,15]. This wide host range yields a variety of pathogen transmission scenarios between the numerous hosts, becoming a concern under an eco-epidemiological point of view.

Various known pathogens have been detected in *H. aegyptium* ticks, such as Rickettsia aeschlimannii and Rickettsia africae [16], *Borrelia burgdorferi* s.l. [17] and Borrelia turcica [18], *Hepatozoon kisrae* [19], *Coxiella burnetii* [20] and *Hemolivia mauritanica* [21]. The last one is the most widely distributed blood parasite of turtles, but its geographical distribution still remains cryptic [22].

Due to the veterinary and medical importance of pathogens transmitted by *H. aegyptium* ticks and their wide host range, ticks from *Testudo graeca* acquired in an animal market in Doha, Qatar, were screened for several pathogens, namely, *Ehrlichia*, *Anaplasma*, *Babesia* and *Hepatozoon/Hemolivia.*

## 2. Materials and Methods

### 2.1. Study Area

A country located on the eastern side of the Arabian Peninsula, Qatar has a desert climate with an arid and hot summer characterized by temperatures ranging between 25 °C and 46 °C. Rainfall is scarce (75.6 mm per year), falling with erratic patterns from October to March. Doha is the country’s capital and its largest city.

### 2.2. Specimen Collection and Processing

#### 2.2.1. Ticks

Ticks included in this study were previously collected and screened for the presence of *Rickettsia* spp. in 2019 [16]. Briefly, a total of 21 ticks were removed from two pet tortoises (*T*. *graeca*), which had been acquired from one of Qatar’s largest animal markets just before presentation at Parkview Pet Center Veterinary Clinic for a health check and ectoparasitic control in May 2018, Doha. The animal market had a total of 20 animal stores, four of which sold tortoises (averaging 10–15 tortoises per store). The removed ticks were previously identified to the species level as *Hyalomma aegyptium* [16] using the morphological criteria already described and further confirmed by PCR using mitochondrial genes (12S and 16S rDNA) as molecular targets [23,24].

#### 2.2.2. Detection of *Ehrlichia*/*Anaplasma*, Babesia and *Hepatozoon*/*Hemolivia* DNA in Ticks

Tick extracted DNA by the alkaline hydrolysis [25] was tested for the presence of *Ehrlichia*, *Anaplasma*, Babesia and *Hepatozoon*/*Hemolivia* by conventional PCR in the Pathology and Immunology Department of the Institute of Biomedical Sciences Abel Salazar, Porto University, according to previously described protocols (Table 1). For PCR, the KAPA HiFi HotStart ReadyMix, KAPA Biosystems (Woburn, MA, USA) was used according to the manufacturer’s instructions. The amplification was performed in Bio-Rad T100^TM^ Thermal Cycler. Aliquots of each PCR product were electrophoresed on 1.5% agarose gel stained with Xpert Green Safe DNA gel stain (Grisp, Porto, Portugal) and examined for the presence of the specific fragment under UV light. DNA fragment size was compared with a standard molecular weight, 100 bp DNA ladder (Grisp, Porto, Portugal). Distilled water was used as negative control.

#### 2.2.3. Sequencing and Phylogenetic Analysis

All *Ehrlichia*-positive and *Hemolivia*-positive amplicons obtained were sequenced for genetic characterization. Amplicons were purified with Exo/SAP Go (Grisp, Porto, Portugal), and bidirectional sequencing was performed with the Sanger method at the genomics core facility of the Institute of Molecular Pathology and Immunology of the University of Porto. Sequence editing and multiple alignments were performed with the BioEdit Sequence Alignment Editor v7.1.9 software package, version 2.1 (Ibis Biosciences). The sequences obtained were subjected to the basic local alignment search tool (BLAST) [29,30,31] using the non-redundant nucleotide database (http://blast.ncbi.nlm.nih.gov/Blast.cgi).

## 3. Results

From the PCR analysis of *H*. *aegyptium* (*n* = 21), three (14.2%) were positive for the *Ehrlichia*/*Anaplasma* 16S rRNA gene, and six (28.6%) were positive for *Hepatozoon* 18S rRNA gene. Bidirectional sequencing and BLAST analysis of consensus sequences of partial 16S rRNA gene of *H*. *aegyptium* tested showed that two shares 99.11% identity with *Candidatus* M. mitochondrii sequences from France (GenBank accession no. EU780455), and one of tested *H*. *aegyptium* presented the highest identity (98.64%) with *Ehrlichia* spp. (GenBank accession no. KX987321) and *E. ewingii* (GenBank accession no. MN148616) sequences from China.

**Figure 1:**
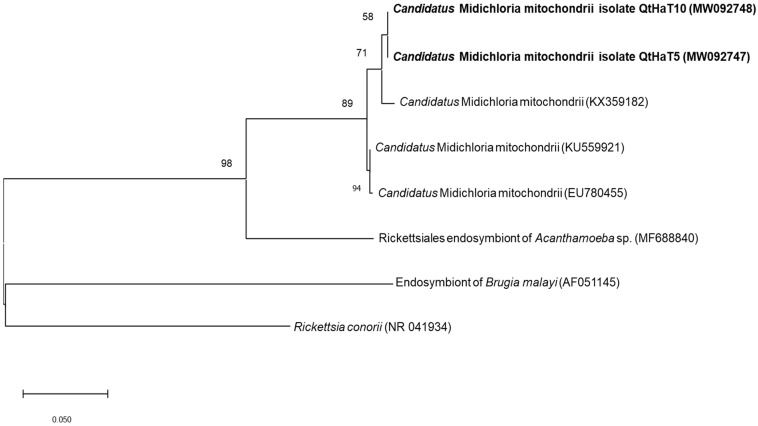
Molecular *Candidatus* M. mitochondrii identification according to phylogenetic analysis using the maximum likelihood method and Tamura-Nei model with the 16S rRNA gene. The analyzed sequences are in bold. The accession numbers for nucleotide sequences from GenBank are presented with species names. The branch numbers mean bootstrap support (1000 replicates). The tree is drawn to scale, with branch lengths measured in the number of substitutions per site.

**Figure 2:**
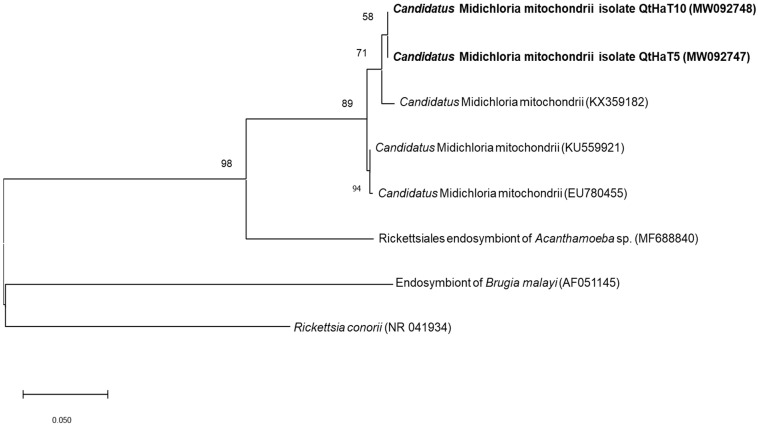
Molecular *Ehrlichia* sp. identification according to phylogenetic analysis using the maximum likelihood method and Hasegawa-Kishino-Yano model with the 16S rRNA gene. The analyzed sequences are in bold. The accession numbers for nucleotide sequences from GenBank are presented with species names. The branch numbers mean bootstrap support (1000 replicates). The tree is drawn to scale, with branch lengths measured in the number of substitutions per site.

Phylogenetic analysis was performed for 16S rRNA sequences to obtain information about their genetic relatedness with other *Candidatus* M. mitochondrii and *Ehrlichia* species. Clustering with reference sequences confirmed the final classification as *Candidatus* M. mitochondrii (Figure 1) and *Ehrlichia ewingii* (Figure 2).

**Figure 3:**
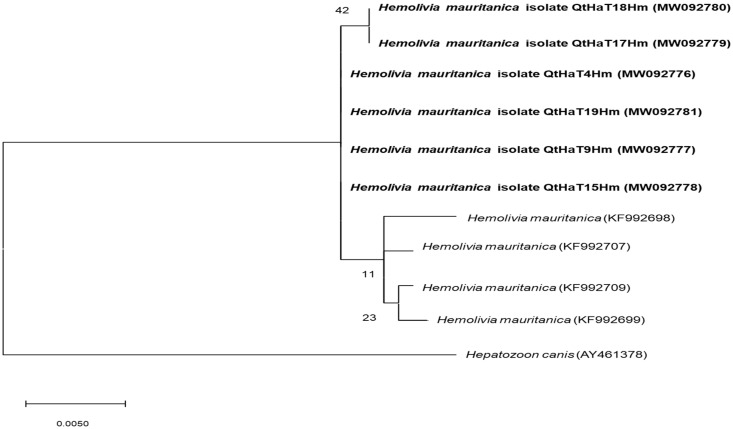
Molecular *Hemolivia mauritanica* identification according to phylogenetic analysis using the maximum likelihood method and Hasegawa-Kishino-Yano model with the 18S rRNA gene. The analyzed sequences are in bold. The accession numbers for nucleotide sequences from GenBank are presented with species names. The branch numbers mean bootstrap support (1000 replicates). The tree is drawn to scale, with branch lengths measured in the number of substitutions per site.

When screening the 21 ticks for the 18S rRNA gene, 5 were found positive for *H. mauritanica*. Further characterization of the 18S rRNA sequences showed a nucleotide identity between 99.70% and 99.84% with *H. mauritanica* sequences from the blood of *Testudo graeca* from Syria (GenBank accession no. KF992707) and Greece (GenBank accession no. KF992710). Phylogenetic analysis was performed for 18S rRNA sequences and confirmed clustering with *H. mauritanica* reference strains (Figure 3).

No amplification was obtained for *Babesia* spp. nor *Hepatozoon* spp. One (4.8%) of the ticks was co-infected with *Ehrlichia* spp. and *H. mauritanica*.

The following accession numbers were assigned to the sequences obtained in this work: MW092747 and MW092748 (16S rRNA gene fragment of *Candidatus* M. mitochondrii), MW092750 (16S rRNA gene fragment of *Ehrlichia* spp.) and MW092776 to MW092781 (18S rRNA gene fragment of *H. mauritanica*).

## 4. Discussion

This report presents the molecular findings for a panel of tick-borne pathogens from a total of 21 *H. aegyptium* ticks previously removed from two *Testudo graeca* tortoises acquired in a large animal market in Doha, Qatar.

In 38% of the 21 *H. aegyptium* collected from *T. graeca* tortoises, tested for *Ehrlichia/Anaplasma*, *Hemolivia/Hepatozoon* and *Babesia* spp., at least one agent was detected. The most commonly detected agent was *H. mauritanica*, with 28.6% of the *H. aegyptum* ticks being positive for it, followed by the endosymbiont *Candidatus* M. mitochondrii, 9.5%, and bacterium *Ehrlichia* spp., 4.8%. *Hemolivia mauritanica* and *Ehrlichia* spp. co-infection was detected in one *H. aegyptium*.

*Hemolivia mauritanica* is a pathogen of tortoises and has *H. aegyptium* as the definitive host [11]. The results obtained in this study are in accordance with previous prevalence levels from Lebanon (38%), Algeria (30.4%) and Bulgaria (14%), but are much lower when compared with results observed in Turkey (82%), Romania (84%), Syria (82%) and Greece (81%) [32].

The molecular analysis of a 345 bp stretch of the 16S rRNA gene showed that a sequence found in a tick presented the highest identity with *Ehrlichia* spp.

*Ehrlichia* spp. are maintained in complex zoonotic systems involving vector ticks and reservoir hosts. These agents affect both humans [33] and animals such as dogs, ruminants [34,35] and even deer [36]. Infected humans [33] and dogs [37] may manifest fever, malaise, leucopenia, thrombocytopenia and abnormal liver function. Tick species that are vectors of these pathogens, such as *Amblyomma*, *Dermacentor*, *Rhipicephalus, Ixodes*, *Haemaphysalis* and *Hyalomma*, also parasitize humans, thus posing a considerable risk [38]. Our results demonstrate a lower occurrence of *H. aegyptium* infected with *Ehrlichia* spp. (4.7%) when compared with recent work, which has shown an occurrence of 30.2% [38].

*Candidatus* M. mitochondrii, an α-proteobacterial symbiont first detected in *Ixodes ricinus*, has a unique intramitochondrial lifestyle [39]. It was the first bacterium shown to reside within the mitochondria and the possible role in ticks is yet to be determined [40]. In the present study, *Candidatus* M. mitochondrii was detected in *H. aegyptium* ticks collected on *T. graeca* from Qatar. As far as we know, this is the first report of the detection of this symbiont in *H. aegyptium* ticks.

Our study reports for the first-time detection of *H. mauritanica*, *Ehrlichia* spp. and *Candidatus* M. mitochondrii in *H. aegytium* ticks collected from pet spur-thighed tortoises, in Qatar, a circumstance which contributes to characterizing the geographical distribution of these agents. The current dimension and growth of international wildlife trade is known not only to act as an avenue for the spread of disease [41] but also poses an important risk to global biodiversity, as well as having an impact on social and economic development [42]. Importation of tick-infested tortoise, later found to be carrying zoonotic pathogens, have been reported in the past [16,43].

## 5. Conclusions

Our study reports for the first-time the detection of *H. mauritanica*, *Ehrlichia* spp. and *Candidatus* M. mitochondrii in *H. aegytium* ticks collected from pet spur-thighed tortoises, in Qatar, a circumstance which contributes to characterizing the geographical distribution of these agents and shows the need of strict surveillance and control to reduce potential non-native diseases while assisting animal conservation.

## Figures and Tables

**Table 1:** Primer sequences used for the detection of tick-borne agents.

Target Gene	Primer Sequence	bp	References
16S rRNA	EHR16SD: 5′-GGTACCYACAGAAGAAGTCC-3′ EHR16SR: 5′-TAGCACTCATCGTTTACAGC-3′	345	[26]
18S rRNA	PIRO-A: 5′-AATACCCAATCCTGACACAGGG-3′ PIRO-B: 5′-TTAAATACGAATGCCCCCAAC-3′	408	[27]
18S rRNA	HEP-F: 5′-ATACATGAGCAAAATCTCAAC-3′ HEP-R: 5′-CTTATTATTCCATGCTGCAG-3′	666	[28]
